# Network Analysis of Metabolite GWAS Hits: Implication of *CPS1* and the Urea Cycle in Weight Maintenance

**DOI:** 10.1371/journal.pone.0150495

**Published:** 2016-03-03

**Authors:** Alice Matone, Marie-Pier Scott-Boyer, Jerome Carayol, Parastoo Fazelzadeh, Gregory Lefebvre, Armand Valsesia, Celine Charon, Jacques Vervoort, Arne Astrup, Wim H. M. Saris, Melissa Morine, Jörg Hager

**Affiliations:** 1 The Microsoft Research—University of Trento Centre for Computational Systems Biology (COSBI), Rovereto, Italy; 2 Nestlé Institute of Health Sciences SA, Lausanne, Switzerland; 3 Nutrition, Metabolism & Genomics group, University of Wageningen, Wageningen, Netherlands; 4 CEA-Genomics Institute- National Genotyping Center, Evry, France; 5 Department of Nutrition, Exercise and Sports, University of Copenhagen, Copenhagen, Denmark; 6 Dept of Human Biology Medical and Health Science Faculty, University of Maastricht, Maastricht, Netherlands; Harbin Medical University, CHINA

## Abstract

**Background and Scope:**

Weight loss success is dependent on the ability to refrain from regaining the lost weight in time. This feature was shown to be largely variable among individuals, and these differences, with their underlying molecular processes, are diverse and not completely elucidated. Altered plasma metabolites concentration could partly explain weight loss maintenance mechanisms. In the present work, a systems biology approach has been applied to investigate the potential mechanisms involved in weight loss maintenance within the Diogenes weight-loss intervention study.

**Methods and Results:**

A genome wide association study identified SNPs associated with plasma glycine levels within the *CPS1* (Carbamoyl-Phosphate Synthase 1) gene (rs10206976, p-value = 4.709e-11 and rs12613336, p-value = 1.368e-08). Furthermore, gene expression in the adipose tissue showed that *CPS1* expression levels were associated with successful weight maintenance and with several SNPs within *CPS1* (cis-eQTL). In order to contextualize these results, a gene-metabolite interaction network of *CPS1* and glycine has been built and analyzed, showing functional enrichment in genes involved in lipid metabolism and one carbon pool by folate pathways.

**Conclusions:**

*CPS1* is the rate-limiting enzyme for the urea cycle, catalyzing carbamoyl phosphate from ammonia and bicarbonate in the mitochondria. Glycine and *CPS1* are connected through the one-carbon pool by the folate pathway and the urea cycle. Furthermore, glycine could be linked to metabolic health and insulin sensitivity through the betaine osmolyte. These considerations, and the results from the present study, highlight a possible role of *CPS1* and related pathways in weight loss maintenance, suggesting that it might be partly genetically determined in humans.

## Introduction

Obesity is a major risk factor for cardiovascular diseases, hypertension, insulin resistance and type 2 diabetes as well as some cancers [1,2]. Multiple studies have shown that weight loss achieved through energy restricted dietary interventions improves the metabolic dysfunctions involved in metabolic syndrome [3–5], and prevents type 2 diabetes. However, the capacity to lose weight and, in particular, to avoid weight regain, shows inter-individual variability that hitherto cannot be explained only by differences in exogenous environmental exposures and lifestyle factors [6,7]. Several studies have shown that this heterogeneity is, at least in part, due to physiological/molecular metabolism differences [8,9]. For example, overfeeding studies have demonstrated a threefold difference in energy efficiency, in turning calories into body mass between individuals [10]. Still, the molecular mechanisms contributing to successful weight loss/maintenance remain poorly understood. Obesity and weight change are complex, and, in order to aid their comprehension, an integrated, multidisciplinary approach is necessary. Here, we show results from combining different types of “omics” data from a large dietary intervention study, with a network analysis approach, in order to elucidate the biological functions involved in the weight maintenance capacity in humans.

Molecular phenotypes can be used as an intermediate to help in identifying relationships between genetic variants and a more global phenotype, such as weight maintenance. For instance, blood metabolite profile can be used to differentiate between obese and lean individuals [11]. For example, plasma glycine levels were negatively correlated with BMI and associated with genetic variants within the carbamoyl-phosphate synthase 1 (*CPS1*) gene, which encodes for a mitochondrial enzyme that catalyzes the synthesis of carbamoyl phosphate from ammonia and bicarbonate [12,13]. These observations raise the possibility that alterations in plasma metabolite concentrations could reflect changes of metabolic processes involved in weight maintenance [14].

The integration of experimental data with systems biology methods could point to possible not yet known pathways to explore [15,16]. With this aim, the utilization of metabolite-protein and protein-protein interaction networks, built from databases, is a powerful way to investigate metabolic pathways potentially correlated with the condition of interest [17]. Networks can allow the consideration of nodes and pathways that would not have been explored with other methodologies. For example, Emilsson et al. [18] constructed an extensive co-expression network on the basis of the human adipose tissue data, to investigate key functional modules associated with obesity, and found that many of the interconnected genes were also associated with the inflammatory response and pathways linked to macrophage activation. In the present work we built a metabolite/protein interaction network of *CPS1* and glycine, in order to investigate whether there could be a link between the pathways in which these are involved, and the complex nature of weight loss and regain, in combination with genetic, metabolomic, transcriptomic and anthropometric data.

The presented results are based on data from one of the worldwide largest dietary intervention study, the Diet, Obesity, and Genes study (DioGenes) [19]. DioGenes is a multicenter, randomized controlled dietary intervention study, involving 8 European countries, which started in 2005. 938 eligible adults underwent an 8-week weight-loss phase following a complete meal replacement low calorie diet (800–1000 kcal/day total energy intake). Subjects who achieved at least 8% weight loss (n = 773) were randomly assigned to one of five weight maintenance arms for 26-weeks. In this paper we will refer as baseline (BL), the initial state (before the study), post-weight loss (PWL) the time after the 8 weeks weight loss period and (PWM) the time point after the 6 months weight maintenance phase. The DioGenes study is unique in its availability of multiple “omics” data from the different time points, including metabolomics, proteomics, adipose tissue RNA expression and genotype data on all participants.

## Materials and Methods

DioGenes was registered in ClinicalTrials.gov (NCT00 390637). Briefly, 932 overweight and obese adults in 8 European centers participated in a dietary program with an 8-week 800–1000 kcal/day whole meal replacement low-caloric diet (LCD; Modifast, Nutrition et Santé, France). Subjects achieving at least 8% of initial body weight loss were randomized to a 6-month ad libitum weight maintenance diet (WMD) consisting in one of four low-fat diets that differed in glycemic index and protein content, or a control diet as described in [20]. Abdominal subcutaneous fat biopsies from the DioGenes protocol were obtained by needle aspiration under local anesthesia after an overnight fast at baseline, at the end of LCD and at the end of WMD. All clinical investigations were performed according to standard operating procedures. Analysis of blood samples was performed at the Department of Clinical Biochemistry, Gentofte University Hospital, Denmark. The study was performed according to the latest version of the Declaration of Helsinki and the Current International Conference on Harmonization (ICH) guidelines. All subjects gave verbal and written informed consent. Ethical approval was obtained from all the regional Ethics Committees of the eight participating centers. The study protocol and informed consent document were approved by the Medical Ethical Committees of Maastricht University and the University of Copenhagen.

### Measurement of targeted blood metabolites

After 12-hour overnight fast, venipuncture was performed in the morning. Fasting blood samples were obtained at each of the 3 CIDs for the analysis of blood metabolites, as also outlined in [20]. NMR measurements were performed by mixing serum samples (100 microliter) with 100 microliter buffer, transferring this to 3 mm NMR tubes and subsequent measurements on a Bruker Avance III NMR spectrometer with cryoprobe. The temperature for the measurements was 37°C and metabolites were determined from 1H J-RES data [21]. The spectra obtained were automatically aligned, phase corrected, base line corrected and integrated. Using the described approach, 36 metabolites could automatically be quantified from the NMR datasets. Eighteen of these metabolites (glucose, acetate, n-acetyl, acetoacetate, proline, glycine, alanine, leucine, isoleucine, valine, tyrosine, creatinine, creatine, lactate, 3-OH-butyrate, hydroxyisobutyrate, oxo-isovaleric acid, urea) passed QC and were included in the GWAS study.

### Genotype data

Genomic DNA was extracted from the buffy coats with a salting out method. Genomic and amplified DNA samples were quality-checked, quantified and normalized to approximately 100 ng/ml and 2.0 mg before genotyping. High throughput SNP genotyping was carried out using the Illumina iScan Genotyping System (Illumina, San Diego, CA, USA). 748 individuals were genotyped using the Illumina 660W-Quad SNP chip. SNP genotyping was done in accordance with manufacturer’s protocols. The Integrated mapping information is based on NCBI’s build 37. Pre-processing of the genotype data was performed with plink (v1.07), which is a free, open-source whole genome association analysis toolset, developed by Shaun Purcell at the Center for Human Genetic Research, Massachusetts General Hospital and Broad Institute of Harvard and MIT [22,23]. The parameters used were mind = 0.01 and geno = 0.01. Furthermore SNPs with MAF < 0.05 and SNPs not in hardy Weinberg equilibrium (p < 10–4) were excluded leading to a set of 456,382 informative SNPs. Pair-wise linkage disequilibrium [24] was calculated with the LDlink web-based application using 1000 Genome phase 3 data (for the CEU population).

### Genome-wide Association Statistics

For association analyses plink software was used. A principal component analysis was performed based on all SNPs after quality control. Principal components were used to control for genetic heterogeneity during association analyses. The number of subjects for which both genotype and metabolite data were available are 641. Linear regression was performed for each SNP for all of the 18 metabolites (NMR) that passed QC considering their level as quantitative traits with gender, age, center and ten first principal components form previous PCA as covariates. This model examines whether each additional allele could alter the trait by equal amounts across the three genotypes. The 5.0e-8 threshold was used to evaluate the significance of each association.

### Networks and gene enrichment

Networks were built from the central nodes glycine and *CPS1*, respectively. Metabolite—gene interactions (glycine targets) were retrieved from Drug Bank 4.2 [25], while protein—protein interactions were retrieved from String 10 [26]. String interactions selection was restricted to the knowledge evidence type, which represents interactions gathered from curated databases. String interactions have a confidence score, which was restricted to above or equal to 0.7, as interactions above this threshold are considered to be in the high confidence range [27]. The networks were built up to 2 neighbors from the central nodes, in order to include a sufficient number of connections to investigate the possible biological pathways relevant to the process under investigation. Networks were built in R library igraph [28], while Cytoscape 3.0 was used for graphical representation. The David online tool [29,30] was used to perform gene pathway enrichment analysis (using KEGG pathways [31]). False discovery rate (FDR) adjusted p-value with a threshold of 0.05 was selected (this corresponded to a false discovery rate of 5%).

### Gene expression

RNA extraction, storage and QC was performed as described in [32]. RNA-seq data from adipose tissues was generated using a pipeline for parallel job submission which consists of RNAStar, version 2.2.0.c [33], with standard parameters, for the alignment process, of samtools, version 0.1.18 [34], for sam/bam file manipulation, and bedtools multicov, version 2.16.2 [35], for computation of transcripts coverage. The genome reference used for mapping was NCBI build 37 (GRCh37).

### eQTL mapping

eQTL analysis in adipose tissue was performed on a subset of 450 individuals from the Diogenes cohort where both the RNA-seq data at time BL and genotype data were available. Pre-processing of the genotype data was performed with plink [22,23] using the same parameters has for the GWAS, leading to a total of 482826 SNPs. The eQTL analysis was performed with the R package MatrixEQTL [36] using a linear model with the following covariates: sex, age and sampling center and 10 first PCs of population stratification. A threshold of adjusted p-value (FDR) < 0.05 was set.

### Statistical methods

The statistical software R 3.0.3 [37] was used for the statistical analysis. A p-value of 0.05 was defined as significance threshold except where otherwise mentioned. The function lm from the stats package in R was used for analyzing the relationship between BMI fold change (between post weight maintenance and post weight loss, computed as (BMI PWM-BMI PWL)/BMI PWL) and gene expression. The same functions were applied to investigate the difference in gene expression between weight maintainers (WMs), defined as subjects which BMI did not change more than 4% during the weight maintenance phase (n = 55) [38], and weight regainers (WRs), whose weight increased by more than 4% (n = 136). Outliers were removed according to the “outlier labeling rule” [39] (k = 1.5), using one iteration, when comparing gene expression in WMs and WRs. The finally compared groups for CPS1 gene expression were of 132 and 53 for WMs and WRs, respectively. S2 Fig shows CPS1 gene expression box plots before and after outliers’ removal. Regressions were corrected for age and sex.

## Results

### GWAS of glycine level

Of the eighteen metabolites analyzed only two showed genome-wide significant associations with SNPs. We identified an association between rs2238732 in the PRODH gene and proline levels. The association of markers in the PRODH gene with proline levels has already been reported by Shin SY et al. [40]. However, neither proline levels nor any of the SNPs in PRODH associated with measure of weight loss/weight maintenance and were not pursued further. We also confirmed the genome-wide association between polymorphisms upstream of *CPS1* gene and blood-derived glycine level in 641 European individuals from the population based DioGenes study [19].

**Fig 1:**
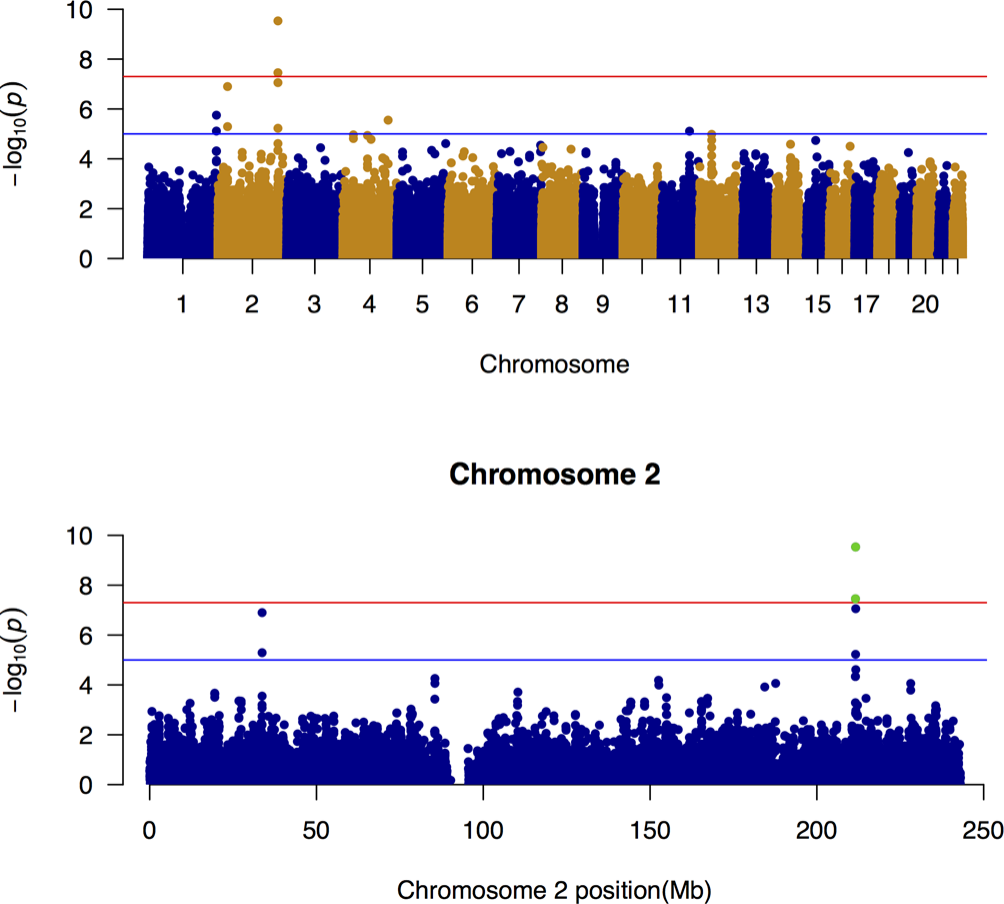
Manhattan plot of Glycine. (a) Genome-wide association of glycine and (b) zoom on chromosome 2. The two green dots represent the SNPs significantly associated with glycine levels: rs10206976 (chr 2:210749914) and rs12613336 (chr 2:210704675), in the *CPS1* gene. Significativity (p—value = 5.0e-8) and suggestive (p—value = 1.0e-5) thresholds are provided as red and blue lines respectively.

These analyses have identified two loci at genome-wide significance (P < 5.0e-8) in *CPS1*: rs10206976 (chr 2:210749914 p-value = 4.709e-11, with allelic odds ratio (OR) = 1.23) and rs12613336 (chr 2:210704675 p-value = 1.368e-08, OR = 1.19) associated with the glycine level in the blood samples (Fig 1). The variant rs1047891 (chr2:210675783), causing the missense mutation Thr1412Asn, was identified as the most probable causal variant using a fine mapping approach [13]. This variant was not in the SNP array used in our study, but is in LD with the SNPs rs10206976 (r = 0.51 D-prime = 1) and rs12613336 (r = 0.5128, D-prime = 0.923).

### *CPS1* eQTL analysis

**Fig 2:**
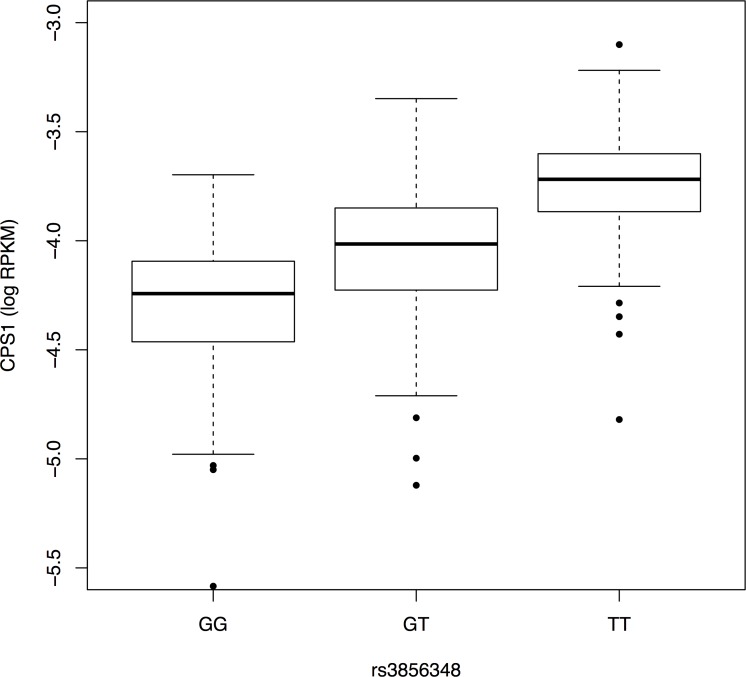
*CPS1* and rs3856348. Correlation between genotypes at rs3856348, and *CPS1* transcription level.

The gene expression profiling from RNA-seq in adipose tissues at baseline for 450 individuals in the DioGenes cohorts were used to find genetic associations with the *CPS1* transcription levels. Genetic mapping of the expression of *CPS1* revealed significant eQTLs (adjusted p-value (FDR) < 0.05). Among those, 17 SNPs were defined as cis-eQTLs since they were located within 100 kbp from the transcription start site of the gene that was measured. Fig 2 shows the association between *CPS1* transcription level and the SNP rs3856348 showing the strongest association (p-value = 2.477e-24). Using the LDlink a web-based application [24], one of these SNPs was found to be in linkage disequilibrium (D-prime > 0.6, R-squared = 0.101) with the glycine associated GWAS SNP rs1047891 (see Table 1).

**Table 1:** Pair-wise linkage disequilibrium.

SNP GWAS	cis-eQTL snp	MAF of GWAS SNP	MAF of cis-eQTL SNP	Distance	RSquared	DPrime
rs1047891	rs7607205	0.2885	0.261	51404	0.101	0.6092
rs1047891	rs2287598	0.2885	0.2951	17344	0.0271	0.5789
rs1047891	rs6725770	0.2885	0.1611	186309	0.032	0.5263
rs1047891	rs6714124	0.2885	0.4732	104442	0.0424	0.3511
rs1047891	rs2371001	0.2885	0.4617	72959	0.036	0.3204
rs1047891	rs2887913	0.2885	0.4141	120300	0.0107	0.2172
rs1047891	rs2012564	0.2885	0.4814	136782	0.0075	0.1798
rs1047891	rs981024	0.2885	0.4105	143827	0.0075	0.1798
rs1047891	rs7573258	0.2885	0.3756	102111	0.0089	0.1398
rs1047891	rs4672587	0.2885	0.3371	3661	0.0108	0.1316
rs1047891	rs2371000	0.2885	0.3644	74727	0.0062	0.1152
rs1047891	rs3856348	0.2885	0.4645	194511	0.0035	0.1023
rs1047891	rs10932334	0.2885	0.4808	199158	0.0027	0.0921
rs1047891	rs3856341	0.2885	0.1062	205860	0.0018	0.0638
rs1047891	rs3732055	0.2885	0.4748	205799	0.0003	0.0289
rs1047891	rs3770685	0.2885	0.4549	201675	0.0002	0.0153
rs1047891	rs2287603	0.2885	0.2047	113533	0	0.0053

Pairwise linkage disequilibrium between the SNP associated to glycine levels and SNPs associated to *CPS1* transcription. The minor allele frequency (MAF) of the GWAS SNP and the cis-eQTL SNPs were taken from dbSNP.

### Glycine and *CPS1* networks

**Fig 3:**
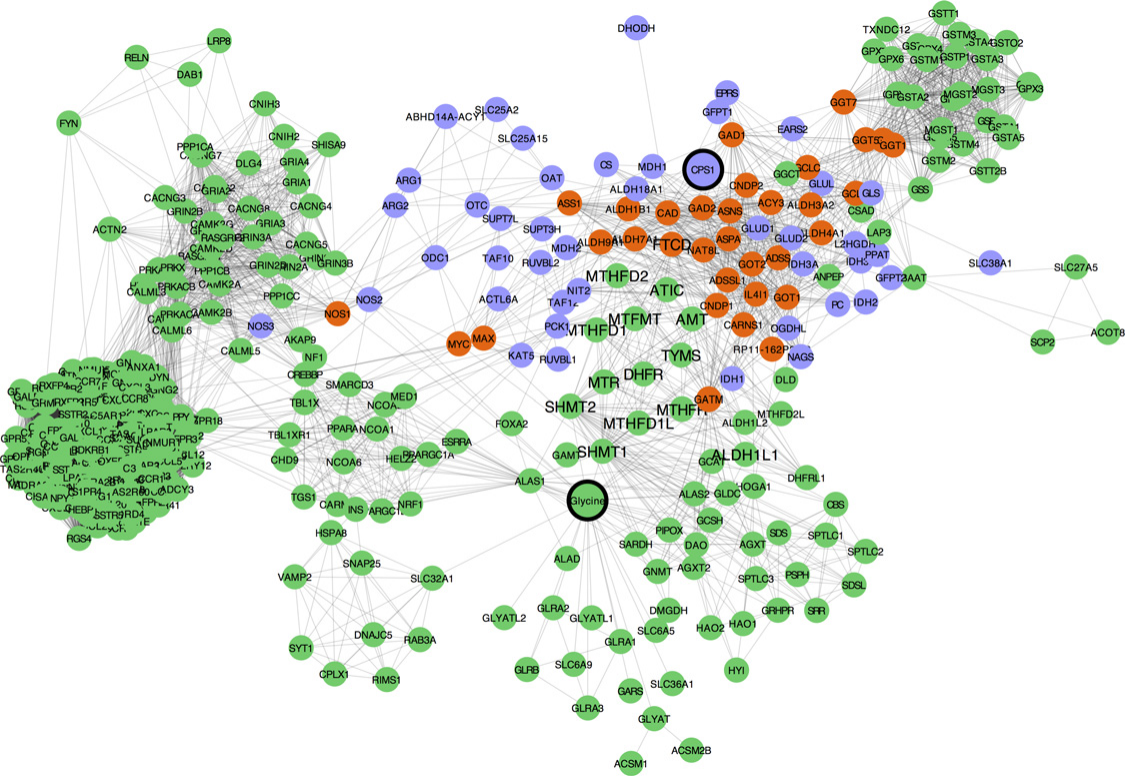
*CPS1* –Glycine network. Networks for *CPS1* and Glycine (77 and 383 nodes, respectively) were built from publicly available databases and subsequently merged. Purple nodes belong to the *CPS1* network; green nodes belong to the Glycine network; orange nodes are shared between the two networks (33). The bigger nodes represent *CPS1* and Glycine. Nodes with bigger labels represent the genes in the One carbon pool by folate KEGG pathway underlined from the gene enrichment analysis.

In order to explore the glycine and *CPS1* connected pathways and to better understand the link between *CPS1* and glycine at the molecular level, a database search was performed to identify protein–protein interactions for *CPS1*, and protein–metabolite interactions for glycine. Findings from these searches were converted into networks, where nodes represent proteins (or glycine), and edges represent protein–protein (or glycine-protein) interactions. The glycine-centered network resulted in 383 nodes and the *CPS1*—centered network in 77 nodes. The merged glycine and *CPS1* network resulted in 427 nodes (33 shared nodes), shown in Fig 3. Gene functional enrichment for the 426 genes in the *CPS1*—glycine network for KEGG pathways [31,41] showed enrichment for 22 terms (adjusted p-value (FDR) < 0.05), reported in S1 Fig. The top pathway for significance is the Neuroactive ligand-receptor interaction (adjusted p-value (FDR) 3.48 e-36). Interestingly, the One carbon pool by folate pathway is represented at the seventh place, with a p-value of 1.45 e-10.

### Weight maintenance and gene expression

**Fig 4:**
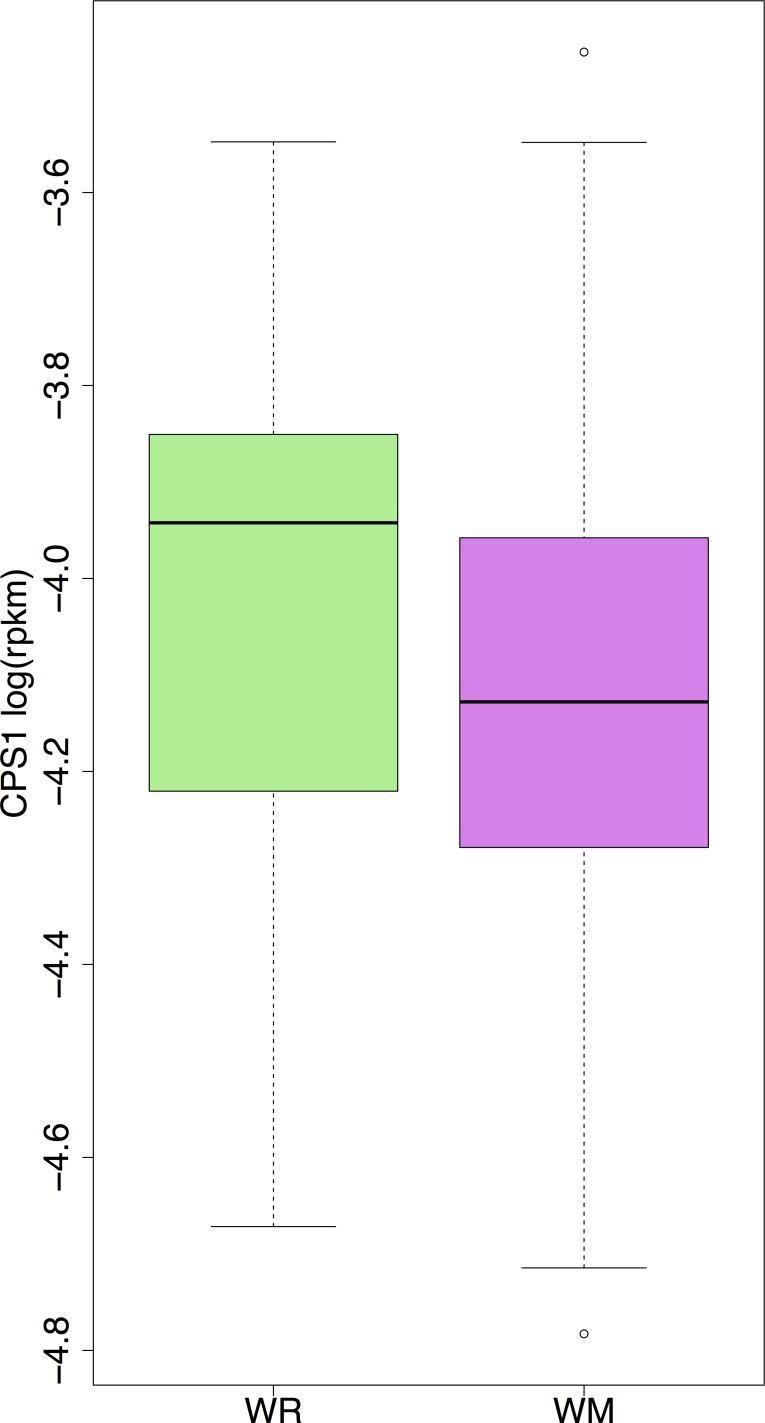
*CPS1* gene expression and weight maintenance. Box plots for *CPS1* gene expression at PWL, for weight maintainers (WMs) and weight maintenance resistors (WRs), defined as ΔBMI < 4% and ΔBMI > = 4% increase (p-value = 0.04, corrected for age and sex).

In order to investigate whether there was a relation between weight maintenance and *CPS1* gene expression, the latter was investigated in successful WMs and WRs individuals. WMs were defined as subjects whose BMI did not vary more than 4% during the weight maintenance phase, while WRs were defined as subjects whose weight increased by more than 4% [38]. *CPS1* gene expression at the beginning of the weight maintenance phase was significantly different between the two groups (Fig 4, p-value = 0.04, correcting for age and sex).

The same analysis was performed for all the genes in the *CPS1*-Glycine network, and the expression value of 45 genes (adjusted p-value (FDR) < 0.1) at the beginning of the weight maintenance phase was significantly different between WMs and WRs. The significant genes and corresponding p-values are reported in Table 2.

**Table 2:** Gene expression and weight maintenance.

n	Gene symbol	p-value	adjusted p-value (FDR)
**1**	*POMC*	7.66E-06	0.00121
**2**	*HRAS*	8.87E-06	0.00121
**3**	*GPX1*	7.14E-06	0.00121
**4**	*CREBBP*	1.50E-05	0.00154
**5**	*TBL1X*	3.05E-05	0.00209
**6**	*NCOA1*	2.97E-05	0.00209
**7**	*GRIN3A*	5.10E-05	0.003
**8**	*RASGRF2*	9.73E-05	0.005
**9**	*TAF10*	1.34E-04	0.00552
**10**	*NCOA2*	1.26E-04	0.00552
**11**	*NF1*	1.66E-04	0.00621
**12**	*MTR*	2.08E-04	0.00712
**13**	*FYN*	2.84E-04	0.00898
**14**	*MED1*	5.62E-04	0.0165
**15**	*CNIH2*	6.25E-04	0.0168
**16**	*MDH2*	6.97E-04	0.0168
**17**	*SPTLC3*	6.52E-04	0.0168
**18**	*NCOA6*	8.10E-04	0.0178
**19**	*LPAR3*	8.22E-04	0.0178
**20**	*GSS*	1.36E-03	0.028
**21**	*GPX4*	1.52E-03	0.0297
**22**	*GRHPR*	1.79E-03	0.0335
**23**	*MGST3*	1.87E-03	0.0335
**24**	*SPTLC2*	3.35E-03	0.0453
**25**	*ACY3*	3.42E-03	0.0453
**26**	*PPARA*	3.39E-03	0.0453
**27**	*ADCY9*	3.46E-03	0.0453
**28**	*SDSL*	2.90E-03	0.0453
**29**	*HCAR1*	2.77E-03	0.0453
**30**	*CCL28*	3.30E-03	0.0453
**31**	*ALDH7A1*	3.53E-03	0.0453
**32**	*CHD9*	3.46E-03	0.0453
**33**	*TAS2R41*	3.78E-03	0.0471
**34**	*GSTK1*	4.04E-03	0.0489
**35**	*GSTP1*	4.39E-03	0.0515
**36**	*CHRM4*	4.62E-03	0.0527
**37**	*BAAT*	5.40E-03	0.06
**38**	*GAL*	6.65E-03	0.0643
**39**	*PPP1CA*	6.72E-03	0.0643
**40**	*AKAP9*	6.44E-03	0.0643
**41**	*GSTO1*	6.45E-03	0.0643
**42**	*MTHFD2L*	6.68E-03	0.0643
**43**	*GFPT1*	6.11E-03	0.0643
**44**	*RUVBL2*	7.46E-03	0.0697
**45**	*GSTM3*	1.07E-02	0.0973

Genes within the *CPS1* glycine network for which expression levels at PWL is significantly different between WMs and WRs, after correction for age and sex (adjusted p-value < 0.1).

Gene expression at the end of the weight maintenance phase of 426 genes within the *CPS1*-Glycine network was analyzed with respect to BMI variation between pre and post weight loss. In a multiple linear regression, the variation in expression of 10 genes was significantly (adjusted p-value (FDR) < 0.1) correlated with BMI variation during weight maintenance (Table 3).

**Table 3:** Gene expression and BMI.

n	Gene symbol	p-value	adjusted p-value (FDR)
**1**	*ANXA1*	0.0007	0.0595
**2**	*APLN*	0.0006	0.0595
**3**	*GLUL*	0.0004	0.0595
**4**	*ALAS1*	0.0003	0.0595
**5**	*GNG2*	0.0007	0.0595
**6**	*GSR*	0.0017	0.0902
**7**	*C3AR1*	0.0016	0.0902
**8**	*CBS*	0.0019	0.0902
**9**	*GARS*	0.0019	0.0902
**10**	*P2RY14*	0.0022	0.0913

Genes within the CPS1 glycine network which expression at PWM correlates with BMI fold change during the weight maintenance phase (adjusted p-value < 0.1, correcting for age and sex).

## Discussion

The results show that genetic variants in *CPS1*, the rate-limiting enzyme of the urea cycle, are strongly associated with plasma glycine levels, and that differences in the expression of *CPS1* suggest a potential causal association with an individual’s capacity to maintain weight after weight loss. This shows the relevance of studying intermediate phenotypes, such as metabolites level, within GWAS results.

In order to insert the results into a broader biological context, building networks helped to identify pathways of interest that would not have been conventionally studied. Glycine is an important metabolite for some key pathways, like the urea cycle, the folate cycle and the one-carbon metabolism. Specifically, glycine provides a precursor pool for betaine, which is an important osmolyte and methyl donor. Betaine has been associated with metabolic, liver and heart health in humans [42]. A high betaine intake has been shown to correlate with higher insulin sensitivity and lower BMI in mice [42,43].

So how may genetic variants and the differences in *CPS1* gene expression contribute to differences in the capacity to maintain weight? One possible model is that individuals who regain weight have a preference to use amino acids and proteins as energy source. They produce more NH4+, which induces *CPS1* expression and leads to more urea. The individuals, who have less amino acid and protein metabolism, use fat as energy source. They therefore produce less NH4+ and *CPS1* is less induced. As a result of higher fat oxidation these individuals may be better at maintaining body weight. This is consistent with the observation that urea levels are higher in weight regainers. In general it has become clear that obese individuals are less flexible in the change from carbohydrate to fat oxidation leading to lower fat oxidation and insulin resistance [44]. The genetic variants influencing *CPS1* gene expression would accentuate this effect and would also explain the observed association between the SNPs and glycine levels in the blood. Individuals with a genotype leading to higher *CPS1* expression have less glycine (at the same input level of NH4+) and the individuals who express less *CPS1* protein have more glycine in the blood. Glycine is a major biochemical mechanism to eliminate ammonia. Glycine conjugating with benzoic acid leads to hippuric acid, which is abundantly found in urine samples.

Variations in *CPS1* quantity and thus the glycine pool may also influence one-carbon metabolism and the bioavailability of betaine. It is noteworthy that the one-carbon metabolism, apart from providing one-carbon bodies for many building blocks in the cell, has been also shown to be linked more directly to mitochondrial function [45] and thus, to energy homeostasis. It might therefore be that the difference in the expression of *CPS1* protein, observed here ultimately impacts energy homeostasis, thereby contributing to the difference in energy efficiency and the capacity to maintain weight after weight loss. To our knowledge this is the first time that such a possible connection has been shown. Our results put a focus on further studies of the relationship between the urea cycle, folate cycle and one-carbon metabolism, on one hand, and energy metabolism on the other. It also provides further evidence that weight maintenance is partly genetically determined in humans [9,46].

The highlighted connections between *CPS1*, glycine and weight maintenance, are further supported by some of the genes in the *CPS1*—glycine network that are correlated with weight maintenance and BMI variation. It is worth of notice as, within the 45 genes which expression differed between weight maintainers and weight regainers (Table 2), some are involved in pathways that could be relevant to weight maintenance. For instance, several genes are involved in fatty acids and lipids metabolism (*PPARA* [47–49], *ALDH7A1* [50,51], *BAAT* [52], *PPP1CA* [53] and *SPTLC2* [54]). Two genes have a role within the one carbon pool by folate pathway, *MTR* [55] and *MTHFD2L* [56]. Furthermore, the following genes are involved in diabetes and insulin signaling: *MDH2* [57], *PPP1CA* [58] and *HRAS* [59]. Also, within the 10 genes which expression significantly correlated with BMI fold change during the weight maintenance phase (Table 3), *GNG2* [60] is involved in diabetes pathways, while *ALAS1* has been shown to be induced in diabetes in mice [30,61].

One main limitation of the present study concerns the need for further experimental verification of the proposed biological meaning of the results. Although different analytical methods have been applied prior to draw the reported conclusions, deeper empirical investigation should be conducted, especially regarding the possible role of the highlighted genes, as well as the unresolved mechanistic links between SNPs and CPS1 expression and CPS1 expression and Glycine.

The use of biological networks from *a priori* knowledge, *i*.*e*. from databases, is useful for data integration and can help biological interpretation of the system by combining complementary viewpoints, each one addressing different aspects of the system. Also, merging a network analysis with independent experimental data can increase data confidence and help interpreting the link with the investigated phenotype, *i*.*e*. weight loss maintenance in this case. This is important especially when studying high-throughput data, which can be affected by noise and incompleteness [62].

## Supporting Information

S1 Fig *CPS1* glycine network KEGG pathways gene enrichment. Gene enrichment performed with David for KEGG pathways. The dendrogram represents the distance within GO terms relatively to the number shared genes. The number of genes in each term is represented in the bar chart while the color of the bars represents the % of pathway coverage (i.e. the ratio of genes in the term in the network over the total number of genes in the term). The p-value for each term’s enrichment is reported in the plot (adjusted p-value (FDR) < 0.05).
(TIF)

S2 Fig *CPS1* gene expression and weight maintenance before (A) and after (B) outliers removal. Box plots for *CPS1* gene expression at PWL, for weight maintainers (WMs) and weight maintenance resistors (WRs), defined as ΔBMI < 4% and ΔBMI > = 4% increase. A) WMs = 137, WRs = 55, p-value = 0.23. B) WMs = 132, WRs = 53, p-value = 0.04. Both regressions were corrected for age and sex.
(TIF)

## Abbreviations

BL: baseline
PWL: post-weight loss
PWM: post-weight maintenance
SNP: Single Nucleotide Polymorphism
WM: weight maintainers
WR: weight regainers
