# Supplementary figures and images for: Network Analysis of Metabolite GWAS Hits: Implication of CPS1 and the Urea Cycle in Weight Maintenance
Source: PLoS One. 2016 Mar 3;11(3):e0150495. doi: 10.1371/journal.pone.0150495 (PMC4777532; doi:10.1371/journal.pone.0150495)

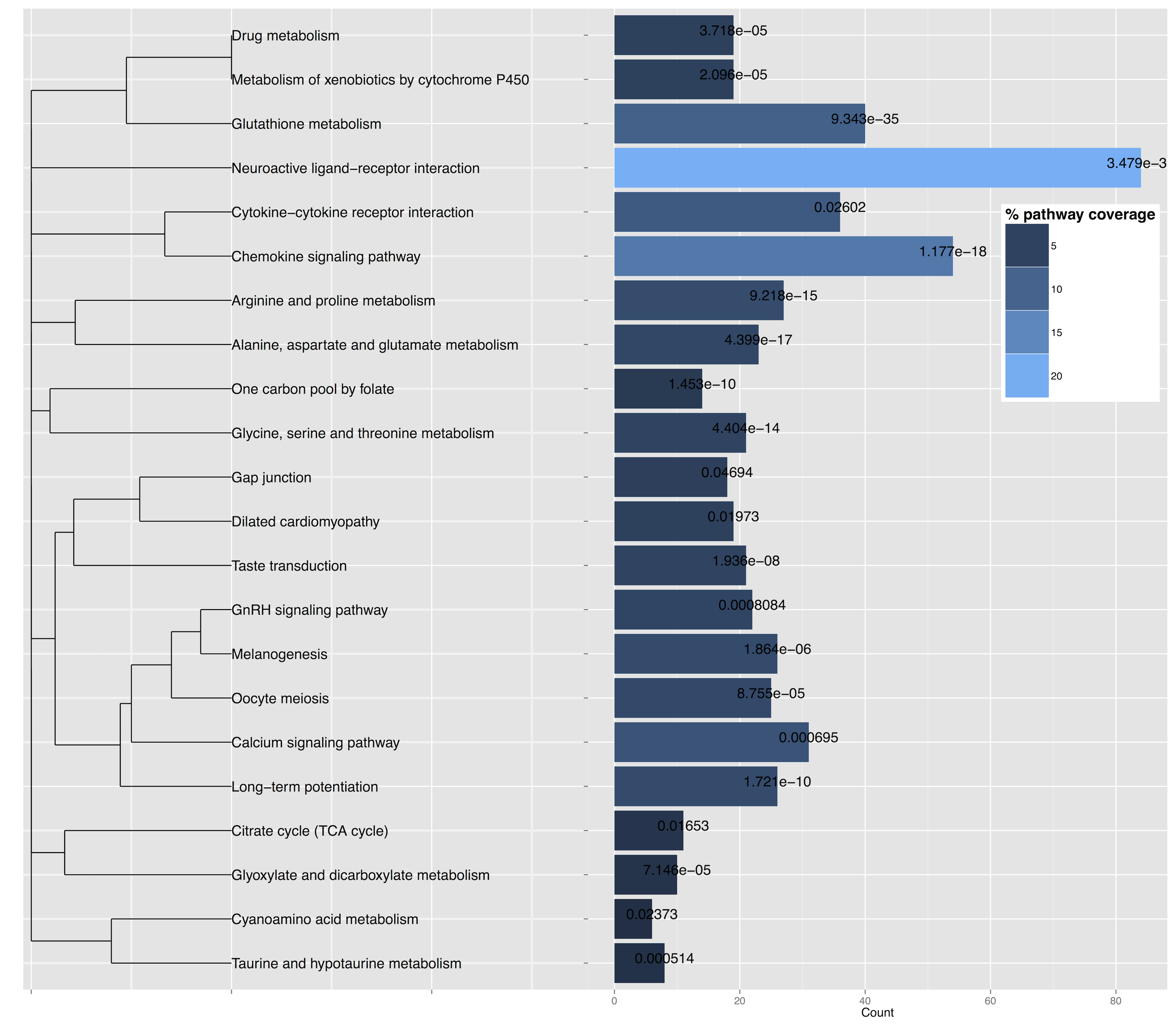

Supplement: S1 Fig — Gene enrichment performed with David for KEGG pathways. The dendrogram represents the distance within GO terms relatively to the number shared genes. The number of genes in each term is represented in the bar chart while the color of the bars represents the % of pathway coverage (i.e. the ratio of genes in the term in the network over the total number of genes in the term). The p-value for each term’s enrichment is reported in the plot (adjusted p-value (FDR) < 0.05). (TIF) [file pone.0150495.s001.tif]

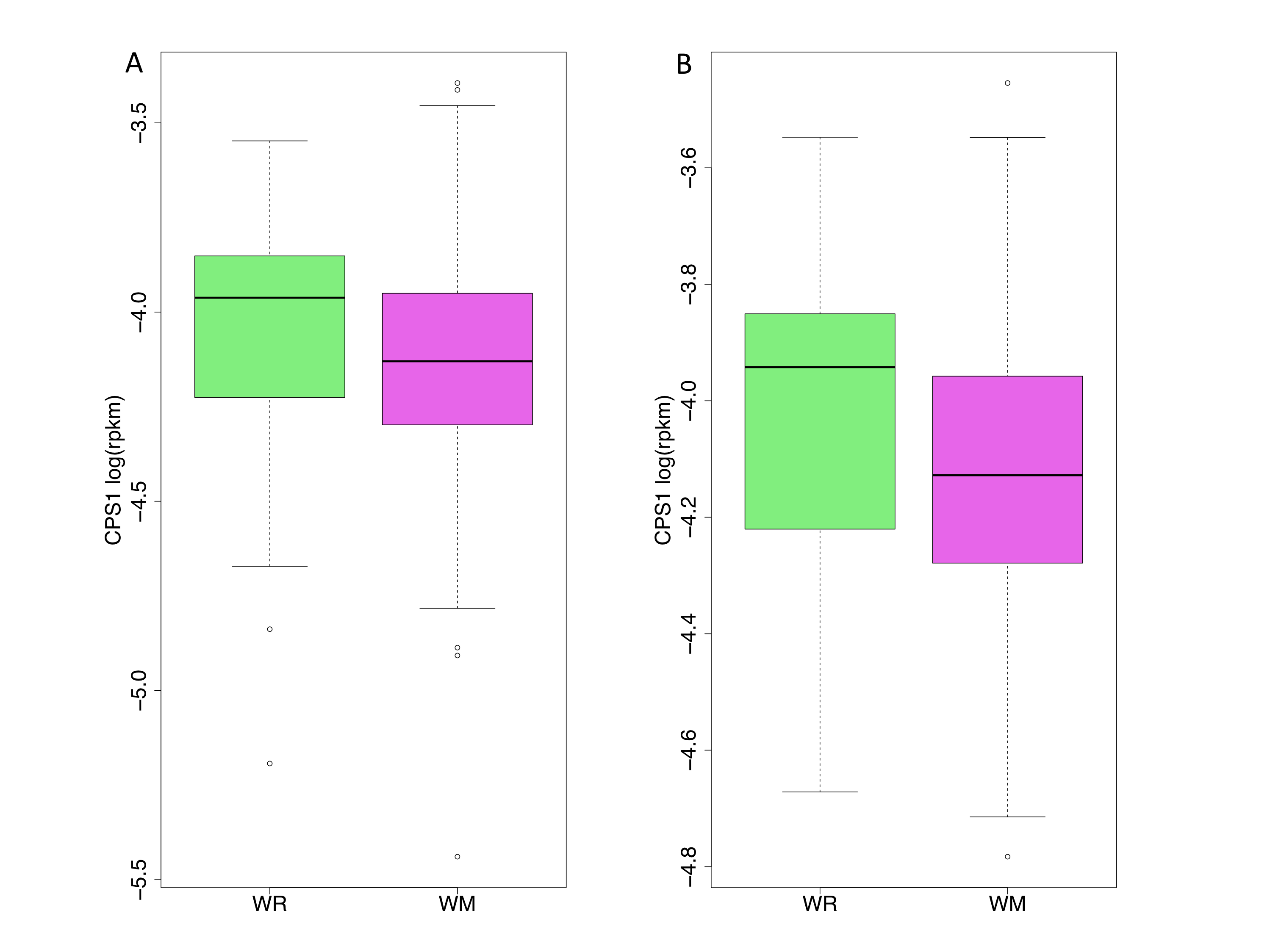

Supplement: S2 Fig — CPS1 gene expression and weight maintenance before (A) and after (B) outliers removal. Box plots for CPS1 gene expression at PWL, for weight maintainers (WMs) and weight maintenance resistors (WRs), defined as ΔBMI < 4% and ΔBMI > = 4% increase. A) WMs = 137, WRs = 55, p-value = 0.23. B) WMs = 132, WRs = 53, p-value = 0.04. Both regressions were corrected for age and sex. (TIF) [file pone.0150495.s002.tif]
